# Replication of the 4p16 Susceptibility Locus in Congenital Heart Disease in Han Chinese Populations

**DOI:** 10.1371/journal.pone.0107411

**Published:** 2014-09-12

**Authors:** Bijun Zhao, Yuan Lin, Jing Xu, Bixian Ni, Min Da, Chenyue Ding, Yuanli Hu, Kai Zhang, Shiwei Yang, Xiaowei Wang, Shiqiang Yu, Yijiang Chen, Xuming Mo, Jiayin Liu, Hongbing Shen, Jiahao Sha, Hongxia Ma

**Affiliations:** 1 Department of Cardiovascular Surgery, Xijing Hospital, The Fourth Military Medical University, Xi’an, China; 2 State Key Laboratory of Reproductive Medicine, Nanjing Medical University, Nanjing, China; 3 Department of Epidemiology and Biostatistics and Key Laboratory of Modern Toxicology of Ministry of Education, School of Public Health, Nanjing Medical University, Nanjing, China; 4 Department of Thoracic and Cardiovascular Surgery, The First Affiliated Hospital of Nanjing Medical University, Nanjing, China; 5 Department of Cardiothoracic Surgery, Nanjing Children’s Hospital, Nanjing Medical University, Nanjing, China; 6 Center of Clinical Reproductive Medicine, First Affiliated Hospital, Nanjing Medical University, Nanjing, China; 7 Department of Cardiology, Nanjing Children's Hospital, Nanjing Medical University, Nanjing, China; 8 Department of Histology and Embryology, Nanjing Medical University, Nanjing, China; Hospital Authority, China

## Abstract

Congenital heart disease (CHD) is the most common form of congenital human birth anomalies and a leading cause of perinatal and infant mortality. Some studies including our published genome-wide association study (GWAS) of CHD have indicated that genetic variants may contribute to the risk of CHD. Recently, Cordell *et al.* published a GWAS of multiple CHD phenotypes in European Caucasians and identified 3 susceptibility loci (rs870142, rs16835979 and rs6824295) for ostium secundum atrial septal defect (ASD) at chromosome 4p16. However, whether these loci at 4p16 confer the predisposition to CHD in Chinese population is unclear. In the current study, we first analyzed the associations between these 3 single nucleotide polymorphisms (SNPs) at 4p16 and CHD risk by using our existing genome-wide scan data and found all of the 3 SNPs showed significant associations with ASD in the same direction as that observed in Cordell’s study, but not with other subtypes- ventricular septal defect (VSD) and ASD combined VSD. As these 3 SNPs were in high linkage disequilibrium (LD) in Chinese population, we selected one SNP with the lowest *P* value in our GWAS scan (rs16835979) to perform a replication study with additional 1,709 CHD cases with multiple phenotypes and 1,962 controls. The significant association was also observed only within the ASD subgroup, which was heterogeneous from other disease groups. In combined GWAS and replication samples, the minor allele of rs16835979 remained significant association with the risk of ASD (OR = 1.22, 95% CI = 1.08–1.38, *P* = 0.001). Our findings suggest that susceptibility loci of ASD identified from Cordell’s European GWAS are generalizable to Chinese population, and such investigation may provide new insights into the roles of genetic variants in the etiology of different CHD phenotypes.

## Introduction

Congenital heart disease (CHD) usually refers to abnormalities in the heart’s structure or function that arise before birth [1]. As the most frequent birth defect, CHD affects about 0.8% of live births [2], [3], and it is also a leading cause of perinatal and infant mortality, causing more than 220,000 deaths globally every year [4]. In China, epidemiological studies suggest an obvious increasing trend of CHD mortality with the overall mortality rate increasing from 141 in 2003 to 229 in 2010 per 10,000,000 person-years [5]. CHD can be classified into three broad categories: cyanotic heart disease, left-sided obstruction defects and septation defects [1]. Among them, septation defects are most common and can affect septation of the atria (atrial septal defect, ASD), septation of the ventricle (ventricular septal defect, VSD) or formation of structures in the central part of the heart (atrioventricular septal defect, AVSD) [1]. CHD may occur as part of recognized chromosomal and Mendelian syndromes [6], [7]; however, it manifests as a non-syndromic, non-Mendelian condition in 80% of cases and may result from a multifactorial inheritance model that involves environmental factors and genetic predisposition [8]. In the past years, several genes linked to CHD have been identified, including T-box transcription factors (*TBX1*, *TBX5* and *TBX20*), homobox transcription factors (*NKX2.5* and *NKX2.6*), basic helix-loop-helix transcription factors (*HAND1* and *HAND2*), and GATA binding protein 4 (*GATA4*) [9].

More recently, genome-wide association studies (GWAS) have emerged as a powerful tool to identify novel susceptibility loci or genes of disease [10]. In May 2013, our research group conducted a GWA scan with 945 patients of ASD, VSD and ASD combined VSD cases and 1,246 non-CHD controls, followed by a two-stage validation with 2,160 ASD, VSD and ASD combined VSD cases and 3,866 controls [11]. We identified 2 loci at 1p12 (rs2474937 near *TBX15* gene, odds ratio (OR)  = 1.40, *P* = 8.44×10^−10^) and 4q31.1 (rs1531070 in *MAML3* gene, OR = 1.40, *P* = 4.99×10^−12^) associated with CHD significantly (*P*<5.0×10^−8^). However, these variants may represent only a very small proportion of SNPs contributing to CHD risk because of the strict criteria of the GWAS significance level (*P* = 10^−7^ or *P* = 10^−8^). Simultaneously, a GWAS of multiple CHD phenotypes in European Caucasians was conducted by Cordell *et al*. In that GWAS, when all CHD phenotypes were considered together, no region achieved genome-wide significant association. However, 3 SNPs were identified to be associated with ASD risk significantly after the stratification analysis was performed in different diagnostic groups (rs870142, *P*_combined_ = 2.61×10^−10^, odds ratio (OR) = 1.456; rs16835979, *P*_combined_ = 2.94×10^−10^, OR = 1.452 and rs6824295, *P*_combined_ = 9.73×10^−10^, OR = 1.437) [12]. However, it is uncertain whether these associations could be applicable to diverse populations. Therefore, we first investigated the association between the 3 SNPs (rs870142, rs16835979 and rs6824295) and CHD risk by using our existing genome-wide scan data and then performed a replication study with additional 1,709 CHD cases with multiple phenotypes and 1,962 controls.

## Materials and Methods

### Ethics Statement

This study was approved by the institutional review board of Nanjing Medical University. The design and performance of current study involving human subjects were clearly described in a research protocol. All participants over the age of 18 would complete the informed consent in writing before taking part in this research. For the participants under the age of 18, parental written consent was obtained.

### Study populations

Study population of GWAS scan has been described previously [11]. For the replication stage, 1,709 CHD cases and 1,962 controls were recruited from the First Affiliated Hospital of Nanjing Medical University and the Affiliated Nanjing Children’s Hospital of Nanjing Medical University, Nanjing, China, between March 2009 and May 2013. CHD were diagnosed based on echocardiography, some of which were further confirmed by cardiac catheterization and/or surgery. Cases that had clinical features of developmental syndromes, multiple major developmental anomalies or known chromosomal abnormalities were excluded. Exclusion criteria also included a positive family history of CHD in a first-degree relative (parents, siblings and children), maternal diabetes mellitus, phenylketonuria, maternal teratogen exposures (e.g., pesticides and organic solvents), and maternal therapeutic drugs exposures during the intrauterine period. Controls were non-CHD outpatients from the same geographic areas. They were recruited from the hospitals listed above during the same time period. Controls with congenital anomalies or cardiac disease were excluded. For each participant, approximately 2-ml whole blood was obtained to extract genomic DNA for genotyping analysis.

### Quality control for GWAS scan and genotyping of replication study

The GWA scan was conducted using Illumina Omni zhonghua chips followed by a systematic quality control before association analysis [11]. In brief, we removed samples with low call rates, ambiguous gender, familial relationships, and extreme heterozygosity rate. We also detected population outliers and stratification using a PCA-based method. Our PCA analysis showed that the cases and controls were genetically matched, and the genomic control inflation factor (λ) was 1.065 [11]. SNPs were excluded if they are not mapped on autosomal chromosomes, or have a call rate <95%, minor allele frequency (MAF)<0.05 or *P*<1.00×10^−5^ for Hardy-Weinberg equilibrium. After quality control procedures, a total of 708,275 SNPs in 945 CHD cases and 1,246 controls were included in the final GWA analysis [11]. The genotyping analyses in the replication study were performed by using the TaqMan allelic discrimination Assay (Applied Biosystems, Inc, USA). The information of the primers and probes were provided in **Table 1**. A series of methods were used to control the quality of genotyping: (i) case and control samples were mixed on each plate; (ii) genotyping was performed without knowing the case or control status; (iii) two water controls were used in each plate as blank control; (iv) five percent of the samples were randomly selected for repeat genotyping. The accurate concordance rate of genotyping for the SNP rs16835979 was 100.00% in the replication stage.

**Table 1:** Primers and probes of rs16835979.

rs16835979	
F-primer	TGTGGACTCTAGAATGGACTTCCA
R-primer	TGTCAGATTAGGACCATCTCCATCT
FAM-probe	FAM-AGTGACTTTACTGTCCTC-MGB
HEX-probe	HEX-AAGTGACTTTAATGTCC-MGB

### Statistical analysis

Population structure was evaluated by PCA as implemented in the software package EIGENSTRAT 3.1. The significant (*P*<0.05) top eigenvector was included in the logistic regression model as a covariate for genetic association analysis [11]. Hardy-Weinberg equilibrium was evaluated using the χ2 test in all of the controls. The genome-wide association analysis was performed using an additive model in a logistic regression analysis in PLINK 1.07 (http://pngu.mgh.harvard.edu/~purcell/plink/). The statistical significance was declared at α  = 0.05. *P* values were two-sided, and ORs were estimated with an additive model by logistic regression analyses. The linkage disequilibrium (LD) analysis of the 3 SNPs (rs870142, rs16835979 and rs6824295) was performed in the controls of GWAS by using PLINK 1.07. Other analyses were performed using Stata version 9.2 (StataCorp LP, TX). The Chi-squared based Cochran’s Q statistic was calculated to test for heterogeneity between groups in stratified analysis. We used inverse-variance-weighted model for meta-analysis when performing combined analysis. Random-effects model was used when heterogeneity between studies exists, that is, *P* value for heterogeneity test was less than 0.05; otherwise, fixed-effect model was used.

## Results

The characteristics of the 945 CHD cases and the 1,246 non-CHD controls in GWA scan have been described previously [11]. Moreover, selected characteristics of the 1,709 cases and 1,962 non-CHD controls in the replication study were shown in **Table 2** and there were similar distributions of age and sex between cases and controls (*P* = 0.221 and 0.870, respectively).

**Table 2:** Characteristics of CHD cases in validation stage.

Variables	Case (N = 1,709)	Control (N = 1,962)	*P*
Age (mean±SD)	3.21±8.70	3.45±7.93	0.221
Sex			
Male	738 (43.2%)	842 (42.9%)	0.870
Female	971 (56.8%)	1,120 (57.1%)	
Phenotype			
ASD	367		
VSD	432		
TOF	211		
Other CHDs	699		

ASD: ostium secundum atrial septal defect; VSD: ventricular septal defect; TOF: tetralogy of fallot; Other CHDs included patent ductus arteriosus, ebstein anomaly, double-chambered right ventricle, bicuspid aortic valve, aortic insufficlency, aortic stenosis, mitral insufficiency, rupture of valsalva sinus aneurysm, etc.

*P*-values were derived from the χ2 test for the categorical variable (sex) and t-test for the continuous variable (age).

The genotype distributions of these 3 SNPs in cases and controls of GWA scan were shown in **Table 3**. The observed genotype frequencies were all in Hardy-Weinberg equilibrium in controls (*P* = 0.337 for rs870142, *P* = 0.370 for rs16835979 and *P* = 0.385 for rs6824295). Logistic regression analyses showed that no obvious associations were found between the 3 SNPs and overall risk of CHD in an additive model (**Table 3**). Then, we performed stratification analysis by different diagnostic groups (ASD, VSD and ASD combined VSD) and found that all 3 SNPs showed significant associations with ASD risk (N = 334 cases) in the same direction as that observed in Cordell’s study (rs870142: OR = 1.24, 95% CI = 1.04–1.49, *P* = 0.016; rs16835979: OR = 1.25, 95% CI = 1.05–1.50, *P* = 0.014; rs6824295: OR = 1.24, 95% CI = 1.04–1.48, *P* = 0.019). However, no significant association was observed in the other two disease groups (**Table 3**).

**Table 3:** Associations of the 3 SNPs with CHD in our GWA scan.

Chr. (cytoband)	Position (bp)	SNP	Study	Cases^b^	Controls^b^	MAF^c^ (Cases)	MAF^c^ (Controls)	OR (95% CI)^d^	*P* d
4p16.2	4698948	rs870142	Combined All (945 cases, 1,246 controls)	116/428/399	145/534/558	0.35	0.33	1.08 (0.95–1.22)	0.259
		G/A^a^	ASD (334 cases, 1,246 controls)	45/164/123	145/534/558	0.38	0.33	**1.24 (1.04–1.49)**	**0.016**
			VSD (534 cases, 1,246 controls)	63/230/241	145/534/558	0.33	0.33	1.00 (0.86–1.17)	0.969
			ASD/VSD (77 cases, 1,246 controls)	8/34/35	145/534/558	0.32	0.33	0.97 (0.69–1.38)	0.876
4p16.2	4686177	rs16835979	Combined All (945 cases, 1,246 controls)	116/426/396	144/536/560	0.35	0.33	1.08 (0.95–1.23)	0.212
		C/A^a^	ASD (334 cases, 1,246 controls)	44/164/121	144/536/560	0.38	0.33	**1.25 (1.05–1.50)**	**0.014**
			VSD (534 cases, 1,246 controls)	64/228/241	144/536/560	0.33	0.33	1.01 (0.87–1.18)	0.896
			ASD/VSD (77 cases, 1,246 controls)	8/34/34	144/536/560	0.33	0.33	1.00 0.70–1.41)	0.986
4p16.2	4665181	rs6824295	Combined All (945 cases, 1,246 controls)	127/445/372	163/554/525	0.37	0.35	1.07 (0.94–1.21)	0.298
		G/A^a^	ASD (334 cases, 1,246 controls)	46/177/111	163/554/525	0.40	0.35	**1.24 (1.04–1.48)**	**0.019**
			VSD (534 cases, 1,246 controls)	71/232/230	163/554/525	0.35	0.35	0.99 (0.85–1.15)	0.878
			ASD/VSD (77 cases, 1,246 controls)	10/36/31	163/554/525	0.36	0.35	1.05 (0.75–1.48)	0.765

a major/minor alleles.

b variant homozygote/heterozygote/wild type homozygote.

c minor allele frequency (MAF).

d OR and *P* were derived from logistic regression analysis in additive model adjusting for the first principal component in GWAS.

As these 3 SNPs were in high LD (*r^2^* = 0.86–0.99) in Chinese population, we selected one SNP with the lowest *P* value in our GWAS scan (rs16835979) to perform a replication study with additional 1,709 CHD cases of multiple phenotypes (ASD: N = 367; VSD: N = 432, Tetralogy of Fallot (TOF): N = 211, other CHDs: N = 699) and 1,962 controls. Similarly, no significant association was observed when all phenotypes were combined together. In the stratification analysis by disease groups, rs16835979 only showed significant association with the risk of ASD (OR = 1.20, 95% CI = 1.02–1.42, *P* = 0.030), which was heterogeneous from the other subgroups significantly (*P* for heterogeneity = 0.046) (**Table 4**). In combined GWAS and replication samples, the minor allele of rs16835979 remained significantly associated with ASD risk (OR = 1.22, 95%CI = 1.08–1.38, *P* = 0.001) (**Table 5**).

**Table 4:** Associations of rs16835979 with CHD in replication study.

Chr.(cytoband)	Position (bp)	SNP	Study	Cases^b^	Controls^b^	MAF^c^(Cases)	MAF^c^(Controls)	OR (95% CI)^d^	*P* d	*P* f
4p16.2	4686177	rs16835979	ASD (367 cases, 1,962 controls)	52/165/139	215/888/859	0.38	0.34	**1.20 (1.02–1.42)**	**0.030**	0.046
		C/A^a^	VSD (432 cases, 1,962 controls)	56/178/187	215/888/859	0.34	0.34	1.04 (0.89–1.22)	0.635	
			TOF (211 cases, 1,962 controls)	23/76/110	215/888/859	0.29	0.34	0.81 (0.65–1.02)	0.070	
			Other CHDs (699 cases, 1,962 controls)	91/312/287	215/888/859	0.36	0.34	1.10 (0.97–1.26)	0.136	
			Combined subjects^e^ (1,709 cases, 1,962 controls)	222/731/723	215/888/859	0.35	0.34	1.05 (0.91–1.20)	0.521	

a major/minor alleles.

b variant homozygote/Heterozygote/Wild type homozygote.

c minor allele frequency (MAF).

d estimated in additive model.

e combined via random-effects meta-analysis.

f *P* values for heterogeneity test between groups.

**Table 5:** Associations of rs16835979 with ASD.

Chr.(cytoband)	Position (bp)	SNP	Study	Cases^b^	Controls^b^	MAF^c^(Cases)	MAF^c^(Controls)	OR (95% CI)^d^	*P* d	*P* e
4p16.2	4686177	rs16835979	**GWAS** (334 ASD cases, 1,246 controls)	44/164/121	144/536/560	0.38	0.33	**1.25 (1.05–1.50)**	**0.014**	0.742
		C/A^a^	**Replication** (367 ASD cases, 1,962 controls)	52/165/139	215/888/859	0.38	0.34	**1.20 (1.02–1.42)**	**0.030**	
			**Combined analysis** (combined via fixed-effects meta-analysis)	96/329/260	359/1424/1419	0.38	0.33	**1.22 (1.08–1.38)**	**0.001**	

a major/minor alleles.

b variant homozygote/heterozygote/wild type homozygote.

c minor allele frequency (MAF).

d estimated in additive model.

e *P* values for heterogeneity test between group.

## Discussion

In this study, we attempted to replicate the 4p16 ASD susceptibility loci in Han Chinese population and found that our results were consistent with the results presented by Cordell *et al* to a certain extent. These findings suggest that the susceptibility loci of diseases may have some similarities among different races and ethnicities.

In Cordell’s study, 3 SNPs (rs870142, rs16835979 and rs6824295) were identified to be significantly associated with ASD risk [12]. We performed a LD analysis among the 3 SNPs by the online software of SNAP (http://www.broadinstitute.org/mpg/snap/ldsearchpw.php) and found strong LD between them (*r* ^2^ = 0.89 to 0.96) in CEU (CEPH- Utah residents with European ancestry). After evaluating the LD between the 3 SNPs in the controls of our GWAS samples, we found that they were also in high LD in Chinese populations (*r^2^* = 0.86–0.99). There were 28 other SNPs in the LD plot of the region containing the 3 mentioned SNPs (chr4∶4665181-4698948) and we found that most SNPs were in high LD with the 3 SNPs in European population, while only about half of them were in high LD with the 3 SNPs in Chinese population. Although we included 701 ASD cases and 3,208 controls in this study (334 cases and 1,246 controls in our GWA scan and 367 ASD cases and 1,962 controls in the replication study), the sample size, especially the case sample size was relatively smaller compared to the original Cordell’s GWAS. After performing multiple testing corrections with the Bonferroni single-step method (corrected α: 0.05/3 = 0.017), we found the SNPs rs870142 and rs16835979 remained association with ASD risk; however, the SNP rs6824295 lost its association with ASD. Given the high LD of the 3 SNPs, Bonferroni correction might be too strict. Additionally, owing to the genetic heterogeneity, the allele frequency of the SNP rs16835979 in both populations was different (Chinese population: MAF = 0.380 in cases, MAF = 0.334 in controls; European population: MAF = 0.314 in cases, MAF = 0.235 in controls) and the effect size in the current study was weaker than that in European population (OR = 1.22 in Chinese population vs. OR = 1.46 in European population). Using Levin’s formula [13], we also found that the population-attributable risk of ASD for the SNP rs16835979 was relatively lower in Chinese (7%) than that in Caucasian (9%).

All 3 SNPs locate within an intron of the noncoding RNA gene *LOC100507266* with unknown function. Meanwhile, they also lie in the 320-kb interval between *STX18* (syntaxin 18) and *MSX1* (msh homeobox 1) and locate upstream of both genes. The long non-coding RNA gene *LOC100507266* is known as *STX18* antisense RNA 1 (STX18-AS1). *STX18* is principally located in the endoplasmic reticulum, and it plays roles in transport between the endoplasmic reticulum and Golgi [14]; however, no data are available for its function in heart development. *MSX1* has been showed to be expressed in mesenchymal structures, including the limb buds, pharyngeal arches, neural crest, and endocardial cushions of the heart [15], [16]. In Cordell’s study, *MSX1* was showed to be expressed in the atrial septum during development, both in mouse and chick [12]. *MSX1* and *MSX2*, which belong to the highly conserved *Nk* family of homeobox genes, display overlapping expression patterns and redundant functions in multiple tissues and organs during vertebrate development [15], [16]. The functions of *Msx1* and *Msx2* in endocardial activation prior to EMT via upregulation of *NFATc1* expression have been documented [15]. In addition, *Msx1* has a well-documented role as both a downstream effector and an upstream regulator of Bmp signaling. It has been implicated in regulating epithelial-mesenchymal transition (EMT) during cardiac valve and septa formation [15], [17]. *Hand1* and *Hand2* are also candidate target genes regulated by *Msx1* and *Msx2* [18]. Since myocardium-specific *Hand1* deficiency leads to thickened atrioventricula (AV) valves, which is associated with impaired valve remodeling [19]–[21], decreasing *Hand1* expression in the *Msx1/2* double mutant AV myocardium may be associated with defects in remodeling of the AV cushions into mature valve leaflets [15]. In the mouse, loss both of Msx1 and Msx2 leads to a severe endocardial cushion and conotruncal defects [15], [18]. In humans, mutations in *MSX1* cause orofacial clefting and tooth agenesis [22], [23]. It is speculated that these 3 SNPs may affect the regulation of above genes and thus have associations with the development of ASD; however, the specific mechanisms of the 3 SNPs affecting the risk of ASD still need to be further investigated.

Like Cordell’s study, we did not observe genome-wide significant associations of 4p16 loci with overall CHD risk in our GWAS as well as replication study. The findings suggested that genetic etiology of CHD could have a considerable degree of phenotypic specificity, which was also identified by some recent studies [24], [25]. For the various phenotypes of CHD originated from different part of heart, we believe different genetic factors are involved in their pathogenic process.

Interestingly, our previous GWAS has identified 2 susceptibility loci (rs2474937 at 1p12 and rs1531070 at 4q31.1) for CHD in Han Chinese. However, these variants may contribute to the risk of CHD through different pathways. As described previously, the SNP rs2474937 may affect the expression of *TBX15*, a member of T-box transcription factors that have key roles in the development of the embryonic mesoderm, including of the heart and skeleton. Another SNP rs1531070 is in a LD region overlapping the *MAML3* (mastermind-like 3) gene and mastermind (Mam) is one of the essential components of Notch signaling pathway. In the current study, we confirmed the ASD-specific association of SNP rs16835979 in Chinese. This SNP may influence *MSX1* (msh homeobox 1), which belongs to the highly conserved *Nk* family of homeobox genes. *MSX1* has functions in endocardial activation prior to epithelial-mesenchymal transition (EMT) as well as Bmp signaling. Thus, the findings in the current study supplemented the existing results of our GWAS.

In conclusion, we present evidence for association between the common SNP rs16835979 at 4p16 and the ASD-specific risk in Han Chinese, which extends Cordell’s results in different populations and suggests that the susceptibility loci of disease indeed have some similarities among different races and ethnicities.
